# Curcumin as a Potential Therapeutic Agent in Certain Cancer Types

**DOI:** 10.7759/cureus.22825

**Published:** 2022-03-03

**Authors:** Anish K Vadukoot, Shabna Mottemmal, Pratikkumar H Vekaria

**Affiliations:** 1 Department of Chemistry, Southern Research, Birmingham, USA; 2 Department of Medicine, Kannur Medical College, Kannur, IND; 3 Internal Medicine, Prisma Health University Medical Group, Greenville, USA

**Keywords:** cell-signaling pathways, molecules, hematological malignancies, cancer, curcumin

## Abstract

Cancer is a devastating disease condition and is the second most common etiology of death globally. After decades of research in the field of hematological malignancies and cellular therapeutics, we are still looking for therapeutic agents with the most efficacies and least toxicities. Curcumin is one of the cancer therapeutic agents that is derived from the *Curcuma longa* (turmeric) plant, and still in vitro and in vivo research is going on to find its beneficial effects on various cancers. Due to its potency to affect multiple targets of different cellular pathways, it is considered a promising agent to tackle various cancers alone or in combination with the existing chemotherapies. This review covers basic properties, mechanism of action, potential targets (molecules and cell-signaling pathways) of curcumin, as well as its effect on various solid and hematological malignancies.

## Introduction and background

Cancer is a life-threatening disease and is one of the leading causes of death in developed countries. It is a chronic disease characterized by deregulated signaling pathways involving angiogenesis, proliferation, and apoptosis [1]. Despite the early diagnosis and increase in therapeutic options, the reported incidence and mortality rate have not reduced in the last 30 years [2]. Continued progress in understanding the molecular signaling pathways that drive cancer progression remains a key factor in early diagnosis and possible treatment. Current treatment options like radiation and chemotherapeutic agents as the backbone for cancer are not very effective and are toxic not only to tumor cells but also to normal cells. In the recent past, several strategies have been developed for targeting specific cancer cells without causing severe side effects to normal cells [3]. Several anticancer compounds have been extracted from plant sources such as *Betula alba*, *Taxus brevifolia*, *Curcuma longa*, *Catharanthus roseus*, *Erythroxylum previllei*, and *Cephalotaxus* species [2]. Among these, curcumin (diferuloylmethane) has no noticeable toxicity and, in combination with existing chemotherapeutic agents, is a superior treatment option for certain cancer types [4]. Curcumin is an important component of the curcuminoids family and is isolated from the rhizome of *Curcuma longa L.* (turmeric) [5]. It’s predominantly grown in South Asia and Indonesia. In a pure crystalline form, curcumin was extracted from the turmeric plant for the first time in 1870 [6]. Commonly used as a food colorant, it is a hydrophobic compound that belongs to a chemical class of polyphenols. Curcumin exhibits keto-enol tautomerism, with the predominance of the keto form in an acidic environment and stable enol form under basic conditions. It is much less soluble in water at acidic and neutral pH but soluble in dimethyl sulfoxide (DMSO), methanol, ethanol, and acetone. The potential applications of curcumin include the prevention and treatment of cancer, anti-inflammatory/antioxidant, and antiangiogenic activities [7-9]. These beneficial effects of curcumin are exerted by modulating signaling molecules, including cytokines, chemokines, transcription factors, adhesion molecules, microRNAs, tumor suppressor genes, etc. [10]. Several studies have shown the antitumor activity of curcumin on breast cancer, prostate cancer, brain cancer, lung cancer, and pancreatic cancer [11]. Despite these beneficial effects, curcumin has limited use due to the poor aqueous solubility, chemical instability, bioavailability, and cellular uptake [12]. These limitations hinder the clinical application of curcumin. Several approaches have been considered for improving its selectivity towards cancer cells [13]. Structural modifications of curcumin have been suggested to increase its bioavailability or enhance stability [14,15]. This review focuses on the clinical effects of curcumin and its role as a drug for breast cancer, lung cancer, prostate cancer, and hematological and other malignancies.

## Review

Curcumin for breast cancer

Breast cancer is commonly seen malignancy in women with a high prevalence in industrialized countries [16]. It is the second most common etiology for cancer-related death across the globe [17]. Despite lumpectomy, radiation therapy, chemotherapy, and endocrine therapy, current therapeutic strategies are perceived as inefficacious because of poor response to the treatment, high relapse rate, and drug resistance [18]. These facts endorse the need for a better understanding of underlying biochemical as well as genetic factors to develop novel and effective therapies for breast cancer [19].

**Figure 1:**
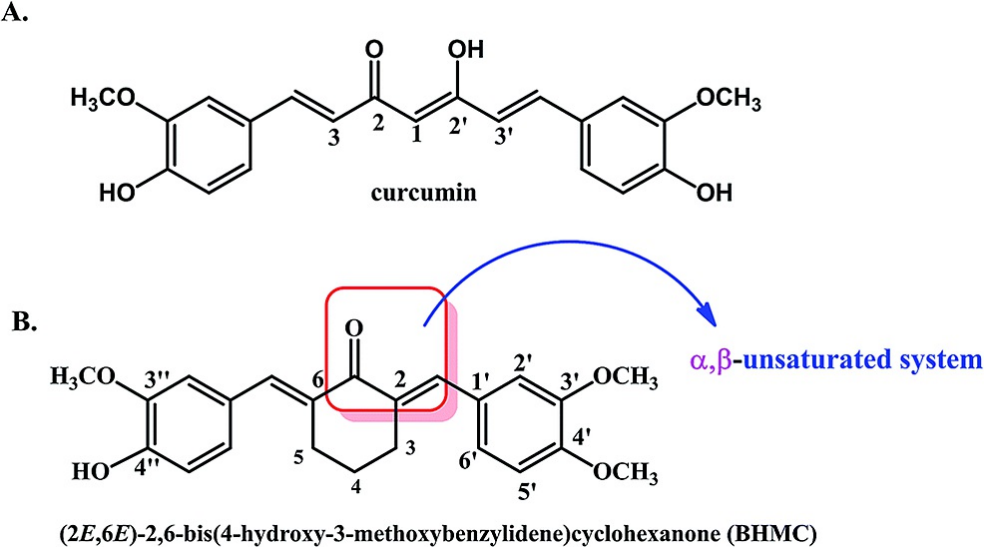
Chemical structure of Curcumin and (2E,6E)-2,6-bis(4-hydroxy-3-methoxy benzylidene) cyclohexanone (BHMC) Reproduced from [37] with permission from the Royal Society of Chemistry.

The proinflammatory transcription factor, nuclear factor-kappa B (NF-κB), plays an important role in the proliferation of breast cancer cells. NF-κB controls the expression of various proteins and more than 500 genes involved in the cell signaling pathways. This is how it contributes to the development of inflammation and cancer [19]. To add, NF‑κB is also considered a valuable marker in breast cancer to show the degree of invasiveness and epithelial‑mesenchymal transition (EMT) [20]. Therefore, the chemical molecules or compounds able to inhibit NF-κB could be utilized in cancer treatment. Curcumin is believed to show its impact on cell growth and invasion of breast cancer partially through the down-regulation of NF-κB signaling pathways [20]. This causes a significant reduction in the expression of chemokine (C‑X‑C motif) ligands 1 and 2 (CXCL1 and CXCL2, inflammatory cytokines) [20], and also leads to modifications in the expression of urokinase plasminogen activator (uPA), uPA receptor, matrix metallopeptidase 9 (MMP‑9), chemokine receptor 4 (CXCR4), and intercellular adhesion molecule 1 [21]. Human epidermal growth factor receptor 2 (HER2) belongs to the epidermal growth factor receptor (EGFR) family and is also a tyrosine kinase (TK) receptor. Overexpression of HER2 induces breast cancer cells proliferation. Due to the overexpression of HER2 in multiple cancer types, it is considered a drug target in cancer treatment [22]. Curcumin may inhibit HER2-TK in breast cancer cell lines alone or in combination with its analogs [23]. Immune-liposome encapsulation helped increase curcumin's selectivity and suppressing action towards HER2 [24]. Cyclin-dependent kinases (CDKs) are threonine/serine kinases that form a protein complex with cyclin partners to help regulate cell cycle progression [25]. Overexpression of deregulated cyclin D1 prompts the aggressive course of breast cancer [26]. Shan Hu et al. reported that curcumin suppressed the proliferation of several breast cancer cell lines, such as MDA-MB-231, MDA-MB-468, T47D, and MCF-7, with a micromolar level of half-maximal inhibitory concentration (IC50) [27]. A study on the mechanism of action of curcumin reveals that it causes cell cycle arrest at the gap-2/mitosis (G2/M) phase by reducing the expression of the cell division control-2 (CDC2) and cell division control-25 (CDC25) proteins while enhancing the p21 expression [27]. The tumor protein P53 or p53 is a well-known activator of apoptosis or cell cycle arrest in response to cellular stress or DNA damage [28]. p53 is a notorious protein causing various cancers by mutating into different human cancer p53. Mutation of p53 causes loss of DNA repair, DNA checkpoints, and cell proliferative control [28]. Recent, next generation sequencing studies confirmed that TP53 mutations are the most frequent genetic alterations in breast carcinomas, reaching 30% of them [29]. Curcumin induces p53-dependent apoptosis and also causes cell cycle arrest in MCF-7 breast cancer cells. In curcumin-treated MCF-7 cells, proapoptotic protein B-cell lymphoma-2 (Bcl-2)-associated X protein (BAX) was found in a high concentration and it indicates curcumin's p53-dependent as well as p53-independent antiproliferative effects [30]. EGFR overexpression is reported to be significantly correlated with large tumor size, poor differentiation, and poor clinical outcomes in breast cancer [31]. Inflammatory and triple-negative breast cancer (TNBC), the most aggressive forms of breast cancer are known to over-express EGFR [32,33]. Xiao-Dong Sun et al. identified that curcumin could inhibit the phosphorylation of extracellular regulated protein kinase (ERK1/2) in MDA-MB-231 cells. ERK1/2 is a major signaling molecule in the downstream pathway of EGFR. This is how curcumin inhibits cell proliferation and induces cell apoptosis, by inhibiting the EGFR pathway in vitro in MDA-MB-231 cells [34]. Phosphatidylinositol 3-kinase/serine/threonine-protein kinase/mammalian target of the rapamycin (PI3K/Akt/mTOR) pathway plays a vital role in cell metabolism, survival, and proliferation. Phosphatidylinositol-4,5-bisphosphate 3-kinase catalytic subunit alpha (PIK3CA) mutations and Akt activation by phosphorylation (pAkt) are often detected in many cancers and especially at high frequencies in breast cancer [35]. Therefore, PI3K/Akt‑mediated signaling pathway is an imperative target for chemotherapies. Yunus Akkoç et al. reported that in metastatic MCF-7 breast cancer cells, overexpression of B-cell lymphoma-2 (Bcl-2) is a constraining factor for curcumin-induced apoptosis [36]. The overexpression of Bcl-2 blocks curcumin-induced autophagy through its inhibitory interaction with Beclin-1 in MCF-7 cells, which is a core component of the Beclin-1/PI3KC3 (phosphatidylinositol 3-kinase catalytic subunit type 3) complex involved in autophagosome nucleation. They found that pre-treatment with LY294002, a PI3K inhibitor, enhanced curcumin-induced autophagy and apoptosis by modifying Bcl-2 expression and subsequent autophagosome formation in MCF-7 breast cancer cells [36]. In vivo effect of curcumin and its derivative (2E,6E)-2,6-bis(4-hydroxy-3-methoxybenzylidene)cyclohexanone (BHMC) had been checked on 4T1 (triple-negative breast cancer cell line) breast cancer cells challenged mice. A study showed that curcumin and BHMC treated mice had low tumor burden, mitotic cells, lung metastasis as well as regeneration capacity compared to the untreated mice [37]. Figure 1 shows the chemical structure of curcumin and BHMC.

Curcumin for lung cancer

Lung cancer is the most common cause of cancer-related death in the world with a five-year survival rate of less than 15%. Lung cancer has traditionally been divided into two major types based on the main histotype, prognostic, and therapeutic implications: small cell lung cancer (SCLC) and non-small cell lung cancer (NSCLC) [38-40]. The majority of cases are NSCLC (85%), of which 10-15% are large cell carcinomas (LCC), 25-30% are squamous cell carcinoma (SCC), and 40% are adenocarcinoma (AC) [41]. EGFR and Kirsten rat sarcoma viral oncogene homolog (KRAS) are two major causal genes known to induce NSCLC. Mutational profiling of 200 lung adenocarcinomas in Korean patients identified mutations of EGFR in 60.5%, KRAS in 12%, and translocations in anaplastic lymphoma kinase (ALK), c-ros oncogene 1 (ROS1), and RET proto-oncogene (RET) in 8.5% of cases [42]. In NSCLC with EGFR mutation, activation of signal transducer and activator of transcription (STAT) and/or Akt is involved in tumor proliferation [43]. Although the involvement of Janus kinase and signal transducer and activator of transcription (JAK ⁄ STAT) signaling in normal lung stem cells is not well known. STAT3, by interacting with SOX2, is reported to contribute to the self-renewal of lung cancer stem cells [44]. NF-­κB is upregulated in lung cancer and preneoplastic lesions, and its activation is correlated with poor prognosis in patients with non-small-cell lung cancer [45]. NF-­κB activation is rapidly induced in response to EGFR oncogene inhibition in lung cancer and promotes resistance to therapy via interleukin 6 (IL­-6) induction [46,47]. The isoenzyme cyclooxygenase 2 (COX-2) is an inducible inflammatory enzyme with increased activity evidenced in lung carcinoma [48]. The therapeutic efficiency of curcumin in lung cancer is exhibited by the suppression of COX-2, EGFR, NF-­κB, and PI3K/Akt signaling pathway. An interesting study by Jeeyun Lee et al. investigated if interferon (IFN)-α stimulation activates an NF-κB in lung cancer cells, and if curcumin annuls IFN-α dependent NF-κB activation and subsequently NF-κB-regulated gene's (cyclooxygenase-2's) expression [49]. The authors found that IFN-α activates NF-κB in A549 lung cancer cells. They reported that the aforementioned hypothesis was correct in the case of A549 lung cancer cells and curcumin effectively down-regulate COX-2 expression through IFN-α-dependent activation of NF-κB [49]. G Radhakrishna Pillai et al. reported that curcumin IC50 of 50 μM is required to induce in vitro apoptosis in A549 cells [50]. Lichuan Wu et al. highlighted the fact that curcumin could inhibit cell proliferation, colony formation, and tumorspheres in lung cancer cell line NCI-H460. The underlying mechanisms of curcumin-induced tumorspheres suppression are mainly due to the inhibition of the JAK2/STAT3 signaling pathway [51]. Furong Liu et al. showed that curcumin exerts a cytotoxic effect on NSCLC A549 cells by inhibiting the PI3K/Akt/mTOR pathway to promote apoptosis and autophagy. It indicates that PI3K/Akt/mTOR signal transduction pathway is a key pathway involved in the role of curcumin in lung cancer [52]. One of the studies showed the effect of curcumin on erlotinib-resistant non-small cell lung cancer (NSCLC) cells. Erlotinib is a known chemotherapeutic agent and it acts via inhibiting an EGFR-tyrosine kinase (EGFR-TK). The combination of erlotinib and curcumin reduced tumor growth remarkably in vivo in erlotinib-resistant NSCLC cells [53]. Ping Chen et al. provided the evidence that gefitinib-resistant NSCLC cells growth could be inhibited by downregulating Sp1/EGFR activity and the receptor tyrosine kinase pathways with the use of curcumin and gefitinib together. This downregulation leads to autophagy-mediated apoptosis and cell death in gefitinib-resistant NSCLC cells. They also validated that curcumin could be utilized, in the treatment of NSCLC with wild-type KRAS and EGFR mutation, as a sensitizer of EGFR-tyrosine kinase inhibitors (EGFR-TKIs) [54].

Curcumin for hematological malignancies

Hematologic malignancies begin in blood-forming tissues that affect the blood, bone marrow, and lymph nodes. Stem cells in the bone marrow develop into white blood cells, red blood cells, or platelets. Blood cancers occur when the uncontrolled growth of abnormal blood cells overtakes the development of normal blood cells and interferes with the regular functions of these cells. It includes various types of leukemia (acute lymphoblastic (ALL), chronic lymphocytic (CLL), acute myeloid (AML), chronic myeloid (CML)), myeloma, and lymphoma (Hodgkin's and non-Hodgkin's (NHL)). The NF-κB pathway is constitutively activated in CLL patients and hence plays a major role in disease development and evolution [55,56]. The JAK and STAT pathway is another active mediator of cytokine signaling in the pathogenesis of solid and hematologic malignancies [57,58]. PI3K/Akt/phosphatase and tensin homolog (PI3K/Akt/PTEN) is another important pathway that is activated in leukemia patients as well as leukemia cell lines together with a decrease in the expression of PTEN gene [59,60]. Several publications showed the involvement of mitogen-activated protein kinases (MAPKs) in the apoptosis of HL-60 cells isolated from patients with human promyelocytic leukemia, one type of acute myeloid leukemia [61]. Cancer cells including human multiple myeloma can induce cells to generate interleukins (IL-1α and IL-1β) or it can directly release IL-1α and IL-1β within the tumor microenvironment [62]. IL-1α and IL-1β can stimulate tumor growth and metastasis via upregulating the expression of angiogenic factors such as IL-8 and vascular endothelial growth factor (VEGF). Chu-Wen Yang et al. investigated the effect and mode of action of curcumin on monocytic leukemia THP-1 cells, derived from human acute monocytic leukemia. The authors demonstrated that curcumin-induced THP-1 cell apoptosis through the activation of c-Jun NH2-terminal kinase/extracellular signal-regulated kinase/activator protein 1 (JNK/ERK/AP1) pathways [63]. Yi-Rong Chen et al. reported that curcumin affects the mitogen-activated protein kinase kinase kinase 1/JNK (MAPKKK1-JNK) pathway by interfering with the signaling molecule(s) like AP-1 and NF-κB as a possible mechanism of action [64]. They speculated that curcumin may affect the JNK pathway by interfering with the signaling molecule(s) at the same level or proximally upstream of the MAPK kinase kinases (MAPKKKs) level. Yaowu Zhang et al. showed curcumin can induce apoptosis in osteosarcoma MG63 cells through the mitochondrial pathway. They reported that the effects of curcumin-induced apoptosis in osteosarcoma cells were associated with caspase-3 activation and reduced the levels of Bcl-2 expression [65]. Jia Rao et al. reported a similar function of curcumin in AML cells. They showed that curcumin down-regulates Bcl-2 and induces apoptosis in daunorubicin (DNR)-insensitive CD34+ AML cell lines and primary CD34+ AML cells. The apoptosis was associated with reduced expression of both Bcl-2 messenger RNA (mRNA) and protein, subsequent loss of matrix metalloproteinase (MMP), and activation of caspase-3 followed by poly ADP-ribose polymerase (PARP) degradation [66]. Seong-Su Han et al. reported that at low concentrations, curcumin inhibited the proliferation of BKS-2, an immature B cell lymphoma, more effectively than that of normal B lymphocytes and caused the apoptosis of BKS-2 cells in a dose- and time-dependent manner [67]. The authors concluded that curcumin downregulated the expression of survival genes early growth response 1 (EGR-1), cellular myelocytomatosis (c-myc), and Bcl-extra large (Bcl-XL) as well as the tumor suppressor gene p53 in B cells as its possible mechanism of action. Shilpa Kuttikrishnan et al. investigated the anticancer potential of curcumin in acute lymphoblastic leukemia (ALL). The authors concluded that curcumin suppresses B-pre-ALL cells' growth and proliferation by inactivation of the PI3K/Akt signaling pathway. Inactivation of these signaling molecules leads to activation of apoptosis via the downregulation of anti-apoptotic proteins including Bcl-2 and X-linked inhibitor of apoptosis protein (XIAP) through reactive oxygen species (ROS) generation [68]. Guo-Hua Zhu et al. reported that curcumin significantly induces apoptosis but also partially suppresses invasion in SHI-1 cells (acute monocytic leukemia cell line) in vitro. Their results from polymerase chain reaction (PCR) and western blotting showed that curcumin increased the FasL mRNA level; inhibited Bcl-2, NF-κB, and ERK expression; and activated p38 MAPKs, JNKs, and caspase-3. These effects were possibly triggered via both intrinsic and extrinsic signaling pathways as mentioned by the authors [69]. Zai-Xin Li et al. studied how curcumin affects the proliferation of the Raji cells of Burkitt's lymphoma. Their biochemical studies showed that cell apoptosis increases through upregulation of Bid (BH3-interacting domain death agonist), cytochrome C, and BAX, while oncogene c-Myc was downregulated after curcumin treatment. Taken together, their results suggested that mitochondrial damage induction was the main mechanism of action of curcumin which led to apoptosis of the Raji cells. In vivo effects of curcumin in the xenograft mouse model showed its effective inhibition of tumor growth. All in all, these results were suggestive of curcumin's growth suppressing effect on Burkitt's lymphoma cells both in vivo and in vitro system [70]. 

Curcumin for prostate cancer

Prostate cancer is the fifth leading cause of mortality and the second most common cancer type in males globally [71]. Its occurrence and mortality rates increased with time. Prostate cancer-related mortality was 150,000 in 1990 which increased to 250,000 in 2010 [72]. There were 1,280,000 new cases and 359,000 deaths were reported across the world from prostate cancer. Ethnicity, old age, family history, and obesity are the risk factors associated with prostate cancer [73]. Prostate cancer is either androgen-sensitive or androgen-insensitive. Androgens bind to the androgen receptor (AR) on the prostate epithelium and promote growth and survival [74]. Surgery, chemotherapy, radiation, and hormonal therapy are the current treatment options available for prostate cancer. Multiple studies have been done to evaluate the anticancer effects of curcumin on androgen-sensitive as well as androgen-resistant prostate cancer cell lines [71]. T Dorai et al., 2000, reported that curcumin can reduce the proliferation rate to 20-30% compared to untreated LNCaP cells (androgen sensitive prostate cancer cell-line) [75]. They also noted that anti-apoptotic proteins Bcl-XL and Bcl-2 were significantly suppressed, while the levels of BAX protein stayed unchanged under the same conditions [75]. Asok Mukhopadhyay et al. suggested that curcumin can cause tumor necrosis factor (TNF)-induced apoptosis by suppressing NF-κB activation in the prostate cancer cell [76]. Similarly, curcumin also affects multiple other proteins and pathways, such as c-Jun/activator protein 1 (AP-1), cyclin D1, CDK-4, phosphatidylinositol 3-kinase (PI3K)/mechanistic target of rapamycin (mTOR)/E-twenty six proto-oncogene 2 (ETS2) pathway to reduce proliferation, cell growth in androgen-sensitive prostate cancer cell lines [77-79]. Studies have also shown the anticancer properties of curcumin on the androgen-insensitive prostate cancer cell lines. DU-145 is one of such androgen-insensitive prostate cancer cell lines. Curcumin-treated DU-145 prostate cancer cells showed reduced expression of NF-κB in paired with less proliferation and increased apoptosis [76]. Curcumin additionally downregulated the expression of nuclear transcription factor activator protein-1 (AP-1), composed of c-Fos and c-JUN. Furthermore, the expression and enzymatic activity of proapoptotic proteins procaspase-8 and procaspase-3 increased, while the expression of anti-apoptotic proteins Bcl-XL and Bcl-2 was hindered [76]. Many studies have analyzed the effects of curcumin treatment in vivo on the mice xenografted with various human prostate cancer cells. Thambi Dorai et al., 2001, studied the effect of curcumin on athymic nude mice implanted with LNCap cells. It showed a significant increase in apoptosis and reduction in proliferation of LNCaP cells demonstrated by the increased pycnotic brown staining nuclei in situ [80].

Curcumin for other cancers

Curcumin has been reported to have pharmacological efficiency towards multiple other cancer types like gastric, colorectal, liver, and osteosarcoma. Xiang Zhou et al. reported that curcumin, in combination with oxaliplatin and 5-fluorouracil (5-FU), exhibited synergistic inhibitory effect in xenograft gastric tumor (BGC-823 cancer cells) via downregulation of Bcl-2 and cleavage of caspase-3 and PARP through upregulation of BAX [81]. Gizem Calibasi-Kocal et al. reported the dose-dependent chemopreventive role of curcumin in HCT-116 and LoVo cells (human colon cancer cell lines) possibly through inhibition of NF-κB and/or activation of caspase-3 and caspase-9 [82]. Biqiong Ren et al. demonstrated the antiproliferative role of curcumin on liver cancer and reported its mechanism of action through inhibition of the heat shock protein 70-toll like receptor 4 (HSP70-TLR4) signaling pathway [83]. Duk Su Lee DS et al. demonstrated curcumin-induced p53 upregulation, cell cycle arrest at gap-1/synthesis (G1/S) and G2/S phase, and caspase-3 activation in human osteosarcoma cells [84]. Curcumin has been reported to possess antiproliferative activity towards fibrosarcoma, a rare malignant tumor of the fibrous connective tissue around the bones. MR Guimarães et al. reported that curcumin was able to inhibit cytokine gene expression in diseased periodontal tissue. They discovered curcumin-induced inactivation of IL-6, and IL-11 in a dose-dependent manner while p38 MAPK was not inhibited [85]. Table 1 shows the major cell signaling pathways and molecules that get affected by curcumin in various cancers. 

**Table 1:** The major cell signaling pathways and molecules that get affected by curcumin in various cancer types NF-kB: nuclear factor kappa B; HER2-TK: human epidermal growth factor receptor 2-tyrosine kinase; EGFR: epidermal growth factor receptor; ERK1/2: extracellular regulated protein kinase 1/2; p53: tumor protein P53; KRAS: Kirsten rat sarcoma viral oncogene homolog; EGFR-TK: epidermal growth factor receptor-tyrosine kinase; PI3K/Akt/mTOR: phosphatidylinositol 3-kinase/serine/threonine-protein kinase/mammalian target of the rapamycin; JAK/STAT: Janus kinase and signal transducer and activator of transcription; COX-2: cyclooxygenase-2; JNK/ERK/AP1: c-Jun NH2-terminal kinase/extracellular signal-regulated kinase/activator protein 1; MAPKKK1-JNK: mitogen-activated protein kinase kinase kinase 1-c-Jun NH(2)-terminal kinase; Bcl-2: B-cell lymphoma-2; p38 MAPK: p38 mitogen-activated protein kinases; PI3K/mTOR/ETS2: phosphatidylinositol 3-kinase/mechanistic target of rapamycin/E-twenty six proto-oncogene 2; HSP70-TLR4: heat shock protein 70-toll like receptor 4; IL-6: interleukin 6; IL-11: interleukin 11.

	Cancer types	Major cell signaling pathways/molecules affected by Curcumin
1.	Breast Cancer	NF-kB, HER2-TK, EGFR, ERK1/2, p53 [19,20,23,30,31]
2.	Lung Cancer	NF-kB, KRAS, EGFR-TK, PI3K/Akt/mTOR, JAK/STAT, COX-2 [49,51-54]
3.	Hematological Cancer	NF-kB, JNK/ERK/AP1, JAK/STAT, MAPKKK1-JNK, Bcl-2, p38-MAPK [63,64,66,69]
4.	Osteosarcoma	Caspace-3, Bcl-2 [65,84]
5.	Prostate Cancer	NF-kB, c-Jun/activator protein 1 (AP-1), cyclin D1, CDK-4, PI3K/mTOR/ETS2 [76-79]
6.	Gastric cancer, colon cancer, liver cancer and fibrosarcoma	Bcl-2, PARP, Caspase-3, NF-kB, Caspase-9, HSP70-TLR4, IL-6, and IL-11 [81-83,85]

Lastly, many experiments were conducted to improve the bioavailability of curcumin to increase its efficacy. Structural modification such as BHMC, as mentioned above in the breast cancer section, is one of the ways to improve its bioavailability, potency, and efficacy. Several other approaches like the use of curcumin nicotinate or PEGylated hyaluronic acid with curcumin have been considered for improving its selectivity towards cancer cells [86]. Chemical/structural modifications of curcumin, including but not limited to the use of adjuvants, nanoparticle encapsulation, liposomes, isomerization, nanogels, and biodegradable micelles, have been recommended to increase its bioavailability or enhance stability [86].

## Conclusions

Curcumin is a potent anti-oxidative, anti-inflammatory, and anti-tumor agent, and it is extracted from rhizomes of *Curcuma *species. Curcumin, as a cancer treatment agent, is recognized to affect multiple targets in different stages of cancer, including angiogenesis, proliferation, metastasis, and apoptosis. The molecular mechanism of action of curcumin has been studied comprehensively. It exerts this effect by interfering with several cell-signaling pathways as well as inducing and inhibiting the production of various growth factors, enzymes, or cytokines. Structural and chemical modifications have been tried to enhance the selectivity, bioavailability, and efficacy of curcumin for cancer. Its therapeutic effects for the breast, lung, prostate, intestinal, liver, and hematological malignancies have been proven in the pre-clinical as well as in vivo studies. Still, curcumin’s effect is questionable under certain circumstances, and its implications for human treatment are considered uncertain. Its poor pharmacokinetic profile and bioavailability lead to its low anticancer potency, which needs to be addressed to improve its therapeutic window. Most of the curcumin formulations are at the proof-of-principal level, and more clinical trials are needed to confirm the effect of curcumin as an anticancer agent. 
